# A double-blind multi-centre randomised placebo-controlled trial of doxycycline in women with recurrent miscarriage and chronic endometritis defined by CD138 positive stromal cells

**DOI:** 10.1093/humrep/deag133

**Published:** 2026-08-20

**Authors:** J Odendaal, N Black, K Fishwick, J Thornton, K Makwana, L Ewington, K Larsen, A Hawkes, C Leeson, J Fisher, R Lall, J Guck, A Coomarasamy, I Granne, M Underwood, G Bouliotis, S Quenby

**Affiliations:** Division of Biomedical Sciences, Clinical Sciences Research Laboratories, Warwick Medical School, University of Warwick, Coventry, UK; University Hospitals Coventry & Warwickshire, Coventry, UK; Division of Biomedical Sciences, Clinical Sciences Research Laboratories, Warwick Medical School, University of Warwick, Coventry, UK; University Hospitals Coventry & Warwickshire, Coventry, UK; Division of Biomedical Sciences, Clinical Sciences Research Laboratories, Warwick Medical School, University of Warwick, Coventry, UK; Division of Biomedical Sciences, Clinical Sciences Research Laboratories, Warwick Medical School, University of Warwick, Coventry, UK; Division of Biomedical Sciences, Clinical Sciences Research Laboratories, Warwick Medical School, University of Warwick, Coventry, UK; Division of Biomedical Sciences, Clinical Sciences Research Laboratories, Warwick Medical School, University of Warwick, Coventry, UK; University Hospitals Coventry & Warwickshire, Coventry, UK; Division of Biomedical Sciences, Clinical Sciences Research Laboratories, Warwick Medical School, University of Warwick, Coventry, UK; Division of Biomedical Sciences, Clinical Sciences Research Laboratories, Warwick Medical School, University of Warwick, Coventry, UK; University Hospitals Coventry & Warwickshire, Coventry, UK; Division of Biomedical Sciences, Clinical Sciences Research Laboratories, Warwick Medical School, University of Warwick, Coventry, UK; University Hospitals Coventry & Warwickshire, Coventry, UK; Warwick Clinical Trials Unit, Warwick Medical School, University of Warwick, Coventry, UK; Warwick Clinical Trials Unit, Warwick Medical School, University of Warwick, Coventry, UK; Warwick Clinical Trials Unit, Warwick Medical School, University of Warwick, Coventry, UK; Tommy’s National Centre for Miscarriage Research, Nuffield Department of Women’s and Reproductive Health, Medical Sciences Division, University of Oxford, Oxford, UK; Nuffield Department of Women’s and Reproductive Health, Medical Sciences Division, University of Oxford, Oxford, UK; University Hospitals Coventry & Warwickshire, Coventry, UK; Warwick Clinical Trials Unit, Warwick Medical School, University of Warwick, Coventry, UK; Warwick Clinical Trials Unit, Warwick Medical School, University of Warwick, Coventry, UK; Division of Biomedical Sciences, Clinical Sciences Research Laboratories, Warwick Medical School, University of Warwick, Coventry, UK; University Hospitals Coventry & Warwickshire, Coventry, UK

**Keywords:** recurrent miscarriage, doxycycline, chronic endometritis, CD138, Bayesian analysis

## Abstract

**STUDY QUESTION:**

Does preconceptual doxycycline prevent miscarriage in women with recurrent miscarriage (RM) and chronic endometritis (CE) defined by CD138+ stromal cells?

**SUMMARY ANSWER:**

Preconceptual doxycycline was not associated with improved livebirth rates in women with CE and a history of RM.

**WHAT IS KNOWN ALREADY:**

CE is characterised by asymptomatic endometrial inflammation and has been associated with RM. Some national and international RM guidelines advise testing and treating for CE with antibiotics, most commonly doxycycline, whereas others do not.

**STUDY DESIGN, SIZE, DURATION:**

Definitions of CE vary widely; in this study, all CD138+ cells, not only morphological plasma cells were included in the assessment. This was an adaptive, multi-centre, parallel-arm, double-blind, placebo-controlled, randomised trial. Recruitment started on January 2020, and follow-up finished on October 2023. Of 2178 women screened, 505 were positive for CD138 and 438 were randomised. A subgroup (202) had repeat endometrial biopsies in the luteal phase of their treatment cycle analysed. A screen negative group (208) were followed up in their first pregnancies. Screen positive women were randomised using an online web tool to preconceptual doxycycline 100 mg or an identical placebo from day 1 of the menstrual cycle, twice daily for 14 days. Patients, their clinicians and outcome assessors were blinded to treatment allocation.

**PARTICIPANTS/MATERIALS, SETTING, METHODS:**

Women, aged ≥18 to <42 years with ≥2 consecutive first trimester miscarriages were recruited in specialist RM clinics from 27 UK sites. Screening was by luteal phase endometrial biopsy with immunohistochemistry for CD138. The primary outcome was cumulative live birth and pregnancies ≥24 + 0 weeks gestation at cessation of the trial. Secondary outcomes included first pregnancy outcome, time to first conception and pregnancy complication rates. A prospective Bayesian analysis with no prior information was undertaken to estimate posterior probabilities.

**MAIN RESULTS AND THE ROLE OF CHANCE:**

There were 113/219 (52%) cumulative livebirths or ongoing pregnancies in the doxycycline group and 109/219 (50%) in the placebo group (adjusted Relative Risk (aRR)1.02; Credible Interval (CrI) 0.85–1.21). Sensitivity analysis did not influence these results. In the CE negative group, there were 62/208 (30%) livebirths or ongoing first pregnancies compared to 89/219 (41%) in the placebo group (aRR 0.73; CrI 0.56–0.95). The amount and pattern of CD138 staining varied equally before and after placebo (98 paired biopsies) and doxycycline (104 paired biopsies) treatments.

**LIMITATIONS, REASONS FOR CAUTION:**

In keeping with the adaptive Bayesian design, the trial was stopped at interim analyses. Whilst a higher-than-expected prevalence of CD138+ CE occurred in this study, a sensitivity analysis increasing the diagnostic threshold did not alter the findings. The second biopsies occurred within the treatment menstrual cycle; hence, we did not exclude a difference in CD138+ CE after menstruation. A few participants obtained additional doxycycline; however, a sensitivity analysis excluding these showed no difference in outcome.

**WIDER IMPLICATIONS OF THE FINDINGS:**

Neither the primary nor secondary outcome measures supported the use of doxycycline in the management of CD138+ CE in this first placebo-controlled trial. Similar differences in CD138 expression before and after placebo or doxycycline suggesting inter-cycle variation may account for previously reported findings.

**FUNDING:**

This study was funded by the Efficacy and Mechanism Evaluation Programme, a Medical Research Council (MRC) and National Institute for Health and Care Research (NIHR) partnership (Funder reference: 17/60/22).

**DISCLOSURES:**

All authors declare no competing interests.

**TRIAL REGISTRATION NUMBER:**

ISRCTN 2394773 https://doi.org/10.1186/ISRCTN23947730

**TRIAL REGISTRATION DATE:**

12 September 2019

**DATE OF FIRST PATIENT’S ENROLMENT:**

3 January 2020

## Introduction

Recurrent miscarriage (RM) has a substantial negative impact on both physical and psychological health of couples (Farren *et al.*, 2021; Quenby *et al.*, 2021). One purported causal factor of RM is the presence of chronic endometritis (CE). This is characterised by asymptomatic inflammation of the endometrium and defined by plasma cell infiltration of the endometrial stroma (McQueen *et al.*, 2015). Currently, the standard diagnostic approach is to use CD138 to highlight plasma cells in the endometrium. Differing patterns and severity of CD138 staining have been reported to be important (Odendaal *et al.*, 2026) with widely varying prevalence of CE (9–84%) in women suffering RM (Cicinelli *et al.*, 2014; McQueen *et al.*, 2014; Liu *et al.*, 2018; Moreno *et al.*, 2018).

CE has been associated with RM in several non-randomised studies; a meta-analysis reported a strong association between CE and RM (OR: 3.59, 95% CI: 2.46–5.24) (Ticconi *et al.*, 2024). It has been hypothesised that CE is caused by a combination of endometrial infections, dysbiosis of the endometrial microbiome and the maternal immune response to these (Dimitriadis *et al.*, 2020; Odendaal *et al.*, 2024).

High resolution rates of histologically defined CE have been reported in non-controlled studies (70–94%) with doxycycline (100 mg twice daily for 14 days) or targeted antibiotic regimens (Johnston-MacAnanny *et al.*, 2010; McQueen *et al.*, 2014; Cicinelli *et al.*, 2015; Kitaya *et al.*, 2017; Song *et al.*, 2021). A systematic review of non-randomised studies found that cured CE was associated with higher ongoing pregnancy or livebirth rate (OR: 1.57; 95% CI: 1.18–2.11). However, no difference was found in livebirth rate in those undergoing antibiotic therapy with a history of RM and CE (OR: 1.68, 95% CI: 0.93–3.03) (Liu *et al.*, 2022). A review of international guidelines identified that screening and treating CE in RM is debated (Hennessy *et al.*, 2021). Some recommend screening and treating CE (Huchon *et al.*, 2016; Toth *et al.*, 2022; Suker *et al.*, 2024), others do not (Practice Committee of the American Society for Reproductive Medicine, 2012a; ESHRE Guideline Group on RPL *et al.*, 2023; Regan *et al.*, 2023; Sierra *et al.*, 2025).

Our multi-centre, placebo-controlled, double-blind adaptive design trial aimed to evaluate the effect of pre-conceptual doxycycline on livebirth rates in women with RM who screened positive for CD138 CE on endometrial biopsy. CE-based diagnosis using CD138 staining varies considerably in the literature (Odendaal *et al.*, 2026). We used permissive study inclusion criteria with sub-stratification for severity of CD138 + CE and planned sensitivity analysis to assess any effect of CD138+ CE severity, age, number of losses or site.

## Materials and methods

### Trial procedures

This was an adaptive, multi-centre, parallel-arm, double-blind, placebo-controlled, randomised, Bayesian trial with a published protocol (Odendaal *et al.*, 2023). The trial was registered 12 September 2019. Ethical approval was obtained from the NHS Research Ethics Committee Northwest-Haydock (19/NW/0462). The study was conducted in compliance with ICH-GCP guidelines and the 2004 Medicine of the Human Use Directive for the clinical trials. The study funders had no role in the design, recruitment, analysis or writing of this report.

Participants were recruited in specialist RM clinics from 27 sites within the United Kingdom (Supplementary Tables S1 and S2). The lead site’s role was twofold: it acted as clinical site for registering eligible local population treated within the site, and a satellite site for recruiting all those eligible across the United Kingdom who were aware of the study and travelled to the site due to lack of local resources to provide timed endometrial biopsy (Supplementary Tables S1 and S2). The biopsies were processed and read in a single laboratory, and interobserver variation monitored by external quality control experts (Supplementary Materials and Methods).

Women aged ≥18 to <42 years with two or more consecutive first trimester losses of an intrauterine pregnancy (<14 weeks gestation) were entered into the screening phase of the trial, the first part of a three-stage consent process (Supplementary Fig. S1). Women were excluded if they had a known treatable cause of RM (antiphospholipid syndrome, uncorrected hypothyroidism, poorly controlled diabetes); experienced a livebirth, stillbirth or termination of pregnancy between or after their last two first trimester miscarriages; were on long-term antibiotic therapy or undertaking antibiotic therapy within the screening menstrual cycle; had a systemic inflammatory condition; or a menstrual cycle length <21 or >42 days. Women were excluded between screening and randomisation if they had taken a course of antibiotics, more than 3 months elapsed or they reported unprotected sexual intercourse.

Endometrial biopsies were performed within a menstrual cycle in which barrier contraception had been used as an outpatient using standard catheters on day 10 ± 4 following ovulation as determined by urinary LH testing. Where a positive ovulation test was not captured, biopsies were performed on day 23 ± 3 of the menstrual cycle.

Samples were processed in a single laboratory with automated immunohistochemistry and external quality control (detailed in Supplementary Materials and Methods). Digital imaging with semi-quantitative methodology was used to assess CD138 staining (detailed in Supplementary Materials and Methods) on whole sections (20–200 mm^2^) (Liu *et al.*, 2018). A CD138+ cell was brown staining that was discreet, consistent and uniformly associated with a nucleus. Glandular cells and background staining were excluded. This approach, which was adapted from Liu *et al.* (2018), utilised more permissive cell inclusion criteria to accommodate the range of the published literature. The CE diagnostic criterion was ≥5 CD138 positive cells per 10 mm^2^. Samples were stratified into four categories: negative (<5 CD138^+^/10mm^2^), mild (5–20 CD138^+^/10 mm^2^), moderate (21–200 CD138^+^/10 mm^2^) and severe (>200 CD138^+^/10mm^2^) (detailed in Supplementary Materials and Methods). The pattern of CD138 staining was recorded as previously described (Odendaal *et al.*, 2026) and the presence of morphological plasma cells was documented. This means that we included all CD138 positive cells and not just those that were morphological plasma cells.

Randomisation was performed utilising an online web tool constructed and hosted by the Warwick Clinical Trials Unit. A 1:1 block allocation was utilised. Minimisation was performed by age (<35 vs ≥35), number of previous miscarriages (≤3 vs >3) and site. Women who were negative for CE were also followed up.

Women were randomised to encapsulated doxycycline 100 mg twice a day for 14 days or an identical placebo capsule from day 1 of the menstrual cycle after randomisation. Both investigational medicinal products (IMPs) were over-encapsulated and packaged to look identical in appearance, colour, shape and weight (Sharp Clinical Services Ltd). Women filled in daily diaries on compliance and unused medication was returned. Treatment non-compliance was defined as missing >3 doses of IMP. The timing of any additional doxycycline taken after randomisation was recorded.

Participants, care givers and outcome assessors were blinded to group assignment.

The primary outcome was defined as livebirth ≥24 + 0 weeks gestation or an ongoing pregnancy ≥24 + 0 weeks gestation at completion of the trial, assessed on a cumulative basis including any pregnancy within the trial period. Secondary outcomes included first pregnancy outcome, pregnancies ongoing at ≥12 + 0 weeks gestation, time to first conception, pregnancy complications, type of miscarriage, ectopic pregnancy and termination of pregnancy.

Women recruited and randomised at the lead site were offered a repeat biopsy. The second sampling was again obtained on day 10 ± 4 after ovulation or day 23 ± 3 of their menstrual cycle, 5–10 days after completing the treatment. CD138 assessment was repeated by observers blinded to the treatment allocation.

The observational cohort of CD138 women had a primary outcome of live birth ≥24 + 0 weeks or ongoing pregnancy ≥24 + 0 weeks gestation in their first pregnancy after biopsy. Secondary outcomes in this cohort were type of pregnancy loss.

Those randomised were followed up 3, 6, 12 and 24 months dated from when medication was started. We continued following up all randomised women until they had a live birth, 2 years post randomisation or study closure, whichever came first. Women who screened negative in the cohort study were followed-up at 3, 6 and 12 months post biopsy or to the first complete pregnancy outcome, whichever came first.

### Statistics

A plausible hypothesis was evaluated for a Bayesian sample size estimation. A baseline incidence of the primary outcome of 48% with an 8% treatment difference was felt to be clinically important. An *a-priori* evaluation of these assumptions at three specified interim stops of 100, 300 and 900 women randomised with a follow-up of no less than 28 weeks was planned. Stopping early for futility (‘No Benefit’) was an option for all three interim analyses but stopping early for efficacy (‘Success’) was allowed at the third interim analysis only. At those milestones, an interim analysis was planned with prespecified stopping criteria:

- Lack of benefit: Posterior probability that intervention is better than control is lower than 0.05.
- Success: Posterior probability that intervention is better than control is higher than 0.995.
- At the final analysis, ‘Success’ was defined as a Posterior probability that intervention is better than control higher than 0.975.

Using these stopping criteria, the study would achieve statistical power of 92% with type I error at 5%. For the ‘Target’ scenario, 43.2% of trials correctly stopped for success and only 0.74% stopped early for No Benefit (type II error). The average sample size under this treatment effect was 1230. For the scenario of ‘No Benefit’, no difference between the arms, 11.2% of trials stopped early for No Benefit (correct decision) and only 0.56% stopped early for Success (type I error, incorrect decision). In this case, the average sample size was 1375.

Categorical variables were summarised as frequencies and percentages whilst continuous variables were summarised as means and standard deviations, if normally distributed, otherwise as medians and interquartile ranges (IQR). Outcomes were analysed according to the Intention-To-Treat (ITT) principle, including all available consented participants. For all discrete outcomes, risk ratios were reported. A similar approach was used for the randomised comparison and the comparison between the placebo group and the CE negative group.

A Bayesian analysis was performed for the primary outcome using a log-binomial generalised linear model with a weakly informative prior, assuming a 50:50 (equal) likelihood of either benefit or harm (RR = 1). The model was adjusted for three minimisation variables: maternal age number of previous miscarriages and site. Treatment effect was summarised with a median risk ratio and corresponding credible interval. The probability of benefit for specific thresholds of interest (2%, 4% and 8%) and harm (0% or below) were additionally reported. It was hypothesised that the use of antibiotic would be justifiable if the positive pregnancy outcome was 8% higher with a posterior probability no less than 97.5%.

Where Bayesian analysis was performed, weakly informative normal prior distributions were used, assuming no prior knowledge of the likelihood of the intervention to have a positive or no effect. The magnitude of the prior is based on its standard deviation and here 1000 was used as standard deviation for those priors. Adaptive Metropolis-Hasting algorithm was utilised to fit the log-Binomial Bayesian models, with two chains and 100 000 iterations per chain, discarding the first 10 000 iterations for the burn-in period. The model’s convergence was examined through trace plots, autocorrelation plots and Gelman Rubin statistics (Supplementary Fig. S2).

As a sensitivity analysis for the primary outcome, the analysis was re-run excluding any protocol violation related to participation time (lost to follow-up), treatment received (sourced doxycycline externally), additional antibiotics or other (relevant) treatments received that might have affected the outcome. As Bayesian statistics were used, the pre-specified subgroup analyses were sensitivity analysis to assess the influence of age (35 vs ≥35), miscarriage history (≤3 vs >3), CE severity or site (largest site vs others).

All analyses were carried out using the ITT set. Secondary outcomes and their differences were summarised using frequencies and chi-squared tests for proportions (unadjusted) and risk ratios for adjusted. As part of the secondary outcomes, the cumulative incidence function was estimated using the Kaplan–Meier method and the curves for the incidence of the conceptions and time to 24 weeks over time for both arms generated.

Within the context of exploratory analyses, a prespecified subgroup using an interaction term was added. In addition, differences between the CE negative cohort and the antibiotic group were assessed. Being aware of the limited power for those analyses, *P*-values are not reported but rather the corresponding 95% confidence intervals are provided. All analyses were performed using Stata v.18 (StataCorp, College Station, TX, USA).

For the repeat sampling sub-study, statistical analysis and graphical output were undertaken with RStudio (Version 2025.05.1 + 513). Continuous variables were assessed for normality and significance testing was performed using paired *t*-tests for normally distributed data or the Wilcoxon rank–sum test for non-normally distributed data. Categorical variables were compared using either the Chi-squared test or Fisher’s exact test when counts were low. A two-sided *P*-value <0.05 was considered statistically significant.

The full trial protocol and statistical analysis plan are available at https://warwick.ac.uk/fac/sci/med/research/ctu/trials/cerm/

## Results

Trial recruitment started on 3 January 2020, was paused due to the SARS-Covid-19 pandemic (19 March 2020–17 September 2020), with ongoing restrictions due to the pandemic substantially affecting recruitment. The first interim analysis, performed when 100 participants had 24-month follow-up, did not reach the futility criteria and no safety concerns emerged. Prior to the planned second interim analysis, we sought additional funding to continue recruitment. In October 2022, the Data Monitoring Comitteee (DMC) was presented with data on the first 306 women who had completed at least 28 weeks from randomisation. They advised that there were no safety issues.

Following meeting of the DMC committee, the summation of the evidence was reviewed. No safety concerns were identified. A holistic review of the trial data, the emerging lack of mechanistic evidence, difficulties in trial recruitment (secondary to Covid-19) and the funder’s reluctance to extend funding led to the conclusion that further recruitment would be futile. The study team remained blind to these findings until after data lock. No additional funding was awarded. Randomisation stopped on 31 October 2022 and follow-up finished on 31 October 2023.

### Trial participants

We screened 2173 women from 27 sites in England, Scotland and Wales (Supplementary Tables S1 and S2) with a history of RPL (Fig. 1); 728 were biopsied resulting in 715 women with interpretable results. Of these, 210 (29%) were CD138 CE negative and 505 (71%), CD138 CE positive; 438 (82.0%) women who screened positive for CE consented to randomisation to either doxycycline (219) or placebo (219). Of the women without CD138 CE, 208 agreed to be followed up. At the lead site, 110 (67%) from the doxycycline arm and 101 (61%) from the placebo arm returned for a second endometrial biopsy, analysable biopsies were obtained from 104 who received doxycycline and 98 that received the placebo.

**Figure 1.**
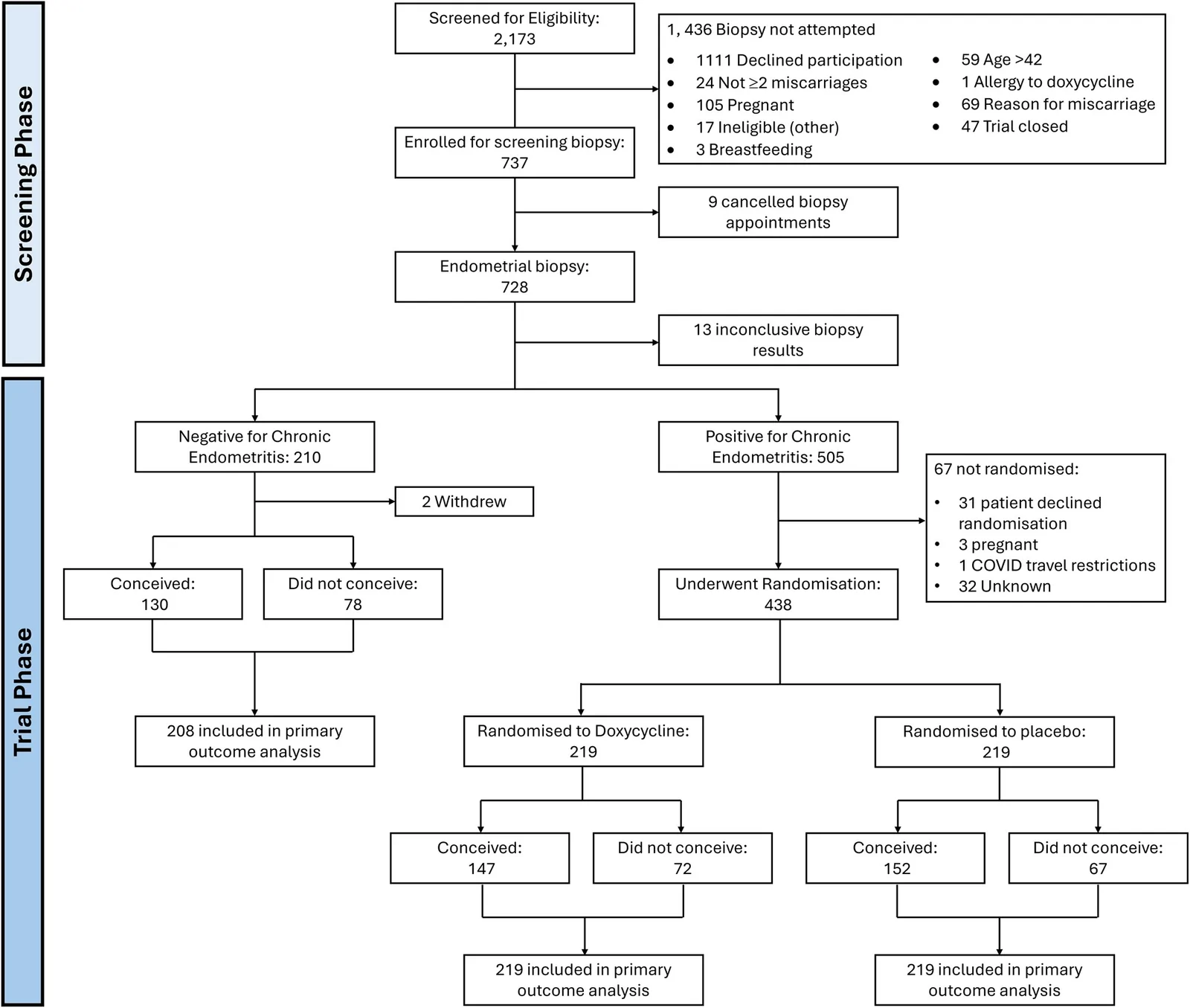
Participant screening, randomisation and outcome.

The baseline characteristics were similar in those randomised to doxycycline and placebo (Table 1) and those CD138 CE negative women in the observational cohort (Supplementary Table S3). The median (IQR) time between biopsy and randomisation was 23 (8) days.

**Table 1.** Participant baseline characteristics.

	Biopsy positive and Randomised		Biopsy negative
	Placebo	Doxycycline	CE-negative
	(n = 219)	(n = 219)	(n = 210)
**Baseline information and characteristics**			
Age mean (95% CI)	34.4(33.8,34.9)	34.9 (34.3,35.5)	34.1 (33.5,34.8)
Age group			
<35yrs, n (%)	109 (49.8)	109 (49.8)	119 (56.7)
≥35, n (%)	110 (50.2)	110 (50.2)	91 (43.3)
BMI, mean (SD)	27.4 (6.1)	27.3 (6.1)	26.6 (5.5)
Smoking status			
Never smoked, n (%)	160 (73.1)	152 (69.4)	139 (66.2)
Ex smoker, n (%)	48 (28.9)	51 (23.3)	48 (22.6)
Current smoker, n (%)	11 (5.0)	16 (17.3)	23 (11.0)
Antibiotics last 3 months			
No, n (%)	201(91.8)	201 (92.2)	195 (92.9)
Yes, n (%)	18 (8.2)	17 (7.8)	15 (7.1)
Attend GUM last 3 months			
No, n (%)	217 (99.1)	218 (99.5)	208 (99.0)
Yes, n (%)	2 (0.9)	1(0.5)	2 (1.0)
Ethnicity			
White, n (%)	186 (84.9)	186 (84.9)	181 (87.0)
South Asian, n (%)	25 (11.4)	22 (10.0)	20 (9.6)
Black/Caribbean, n (%)	3 (1.3)	1	2 (0.9)
Mixed race, n (%)	0	2 (0.9)	2 (0.9)
Other, n (%)	5 (2.3)	8 (3.7)	5 (2.4)
**Past pregnancy history information**			
Live births			
None	130 (59.4)	129 (58.9)	124 (59)
One	66 (30.1)	71 (32.4)	75 (35.7)
More than one	23 (10.5)	19 (8.7)	11 (5.2)
Early miscarriages (<14 WK)			
1 to 3	132 (60.3)	139 (63.5)	112 (53.3)
4 or more	87 (39.7)	80 (36.5)	98 (46.7)
Late miscarriages (wk 14-24)			
None	202 (92.2)	204 (93.2)	202 (96.2)
One	14 (6.4)	15 (6.8)	7 (3.3)
More than one	3 (1.4)	0	1 (0.5)
Ectopic pregnancies			
None	203 (92.7)	197 (90.4)	190 (90.5)
One	15 (6.8)	18 (8.2)	19 (9.0)
More than one	1 (0.5)	3 (1.4)	1 (0.5)
Molar			
None	217 (99.1)	217 (99.1)	207 (98.6)
one	2 (0.9)	2 (0.9)	3 (1.4)
Resolved PUL*			
None	219 (100)	218 (99.5)	210 (100)
One	0	1 (0.5)	0
Termination			
None	207 (94.5)	207 (94.5)	192 (91.4)
One	12 (5.5)	12 (5.5)	18 (8.6)
Stillbirth			
None	218 (99.5)	216 (98.6)	209 (99.5)
One	1 (0.5)	3 (1.4)	1 (0.5)

* PUL, Pregnancy of unknown location.

CE, chronic endometritis; GUM, Genitourinary Medicine clinic.

Of the 438 randomised women, 417 (95%) met the criterion for *treatment compliance* (Supplementary Table S4). The primary reason for non-compliance was lack of collection of the trial medication (n = 8 in each arm). Some women (n = 17) received doxycycline external to the trial; of these, four in each arm received the doxycycline before the first pregnancy outcome, six women received doxycycline after the first pregnancy outcome (Supplementary Table S4). Concomitant medication use was similar in each arm (Supplementary Table S5).

Women who were CD138 CE positive were followed up for a median of 525 (IQR 196–854) days and those CD138 CE negative for 330 (IQR 180–480) days post-randomisation. No difference in length of follow-up was seen across the randomised groups (516 vs. 526 days). The length of follow-up to the first pregnancy outcome was similar in CE positive and negative.

### Outcomes

Of 438 randomised women, 299 (68%) achieved a first pregnancy (Table 2). There were a further 75 second, third and fourth pregnancies; 374 in total. Of these, 316 (84%) were conceived naturally. No difference was seen in the primary outcome (cumulative live birth or ongoing pregnancy ≥24 + 0 weeks gestation) with 113/219 (52%) in the doxycycline group and 109/219 (50%) in the placebo group (Adjusted Relative Risk [aRR] 1.02; credible interval (CrI) 0.85–1.21) (Table 2, Supplementary Fig. S2). There was a low probability of moderate effect on our primary outcome of live births and ongoing pregnancies (0.5–9.9% effect, *Prob.* = 0.34) and a low probability of harm (<1% effect, *Prob. =* 0.36) (Table 3) (Supplementary Fig. S3).

**Table 2.** Pregnancy outcomes.

	Biopsy positive		Biopsy negative		
	Randomised to Placebo	Randomised to Doxycycline		Placebo v Doxycycline aRR (Cr intervals)	Placebo v negative aRR (Cr intervals)
Number	219	219	208	Bayesian estimates adjusted for number of past miscarriages, age group, study site, median risk ratio and corresponding credible interval	
**First pregnancy**					
Conceptions *	152 (69.4)	147 (67.1%)	130 (62.5%)	0.96 (0.85-1.09)	0.68 (0.44-1.02)
Early miscarriage <14	55	54	65	0.87 (0.62-1.21)	1.47 (1.14-1.93)
Ectopic	4 **	1 **	2		
TOP	1	0	0		
Late miscarriage	3	1	1		
Live birth	79	85	57		
Ongoing pregnancy *	10	6	5		
First pregnancy live birth + Ongoing pregnancy	89 (40.6%)	91 (41.6%)	62 (29.8%)	1.00 (0.80-1.23)	0.73 (0.56-0.95)
**Second pregnancy**					
Conceptions	30	33			
Early miscarriage <14	12	11			
Ectopic	2 ***	0			
TOP	0	0			
Late miscarriage	0	0			
Live birth	12	15			
Ongoing pregnancy****	4	5			
Ongoing pregnancy <20 weeks*****	0	2			
**Third pregnancy**					
Conceptions	7	4			
Early miscarriage <14	2	2			
Ectopic	1	0			
TOP	0	0			
Late miscarriage	0	0			
Live birth	3	0			
Ongoing pregnancy****	1	2			
**Fourth pregnancy**					
Conceptions	0	1			
Early miscarriage <14	0	1			
Ectopic	0	0			
TOP	0	0			
Late miscarriage	0	0			
Live birth	0	0			
Ongoing pregnancy****	0	0			
**Total**					
Ectopic pregnancies	7	1		76.7 (1.38–241)	
Miscarriages	72	69		0.90 (0.67–1.22)	
Ongoing pregnancy*	15	13			
Live birth	94	100			
**Cumulative primary outcome**	109 (49.7%)	113 (51.5%)		1.02 (0.85–1.21)	

* Reasons for non-conception include subfertility, 8 stopped trying to conceive and 17 lost to follow-up.

** One had a previous ectopic pregnancy.

*** One had a second ectopic pregnancy.

**** Pregnancy viable at 20 weeks anomalies scan and still viable at time of database lock.

***** Pregnancy viable at 12 week booking scan and still viable at time of database lock.

aRR, adjusted relative risk; Cr, credible; TOP, termination of pregnancy.

**Table 3.** Primary outcome Bayesian regression analysis.

	Risk ratio	95% credible interval	Probability of large effect >10%	Probability of moderate effect 0.5–9.9%	Probability of harm <1%
**Primary analysis**					
Intention to Treat	1.02	0.86-1.22	0.14	0.34	0.36
Per-protocol	1.05	0.87-1.26	0.19	0.35	0.28
**Sensitivity analysis**					
Aged ≥35	1.22	0.84-1.80	0.16	0.13	0.11
>3 Miscarriages	0.93	0.63-1.33	0.09	0.15	0.43
CE severity	1.05	0.85-1.33	0.04	0.11	0.51
Largest site	1.18	0.69- 1.97	0.26	0.47	0.22

CE, chronic endometritis.

There was no difference in time to first conception (Supplementary Fig. S4). In addition, there was no difference in time to 24 weeks gestation (Supplementary Fig. S5). No difference was seen in the cumulative number of miscarriages in the doxycycline group (69/219, 32%) and in the placebo group (72/219, 33%) (aRR 0.90; CrI 0.67–1.22); a similar pattern of type of miscarriage was seen in each group (Supplementary Table S6). There were seven ectopic pregnancies in the placebo group and one in the doxycycline group (aRR 76.7; CrI 0.38–241).

A per-protocol sensitivity analysis excluding known treatment non-compliance additionally showed no significant difference in the primary outcome (aRR 1.05; CrI 0.87–1.26). Sensitivity analyses found no influence of age (35 vs ≥35), miscarriage history (≤3 vs >3), CD138+ CE severity or site (largest site vs others) (Table 3).

In the first pregnancy after randomisation, there was no difference in the live birth and ongoing pregnancy ≥24 + 0 weeks in those randomised to doxycycline or placebo (aRR 1.00; CrI 0.80–1.23). In the first follow-up pregnancy, those negative for CD138+ CE compared to those with CD138+ CE on placebo had significantly fewer live births and ongoing pregnancies 62/208 (29.8%) vs 89/219 (40.6%) (aRR 0.73; CrI 0.56–0.91) and significantly more miscarriages 66/208 (31.7%) vs 55/219 (25.1%) (aRR 1.47; CrI 1.14–1.93) (Table 2).

On the repeat biopsy following treatment, 31.7% (33/104) of the doxycycline group and 26.5% (26/98) of the placebo group tested negative for CD138+ CE; this difference was not statistically significant (*P* = 0.44, Fisher’s exact). There was no difference in the severity or pattern of CD138+ CE staining before and after doxycycline compared to placebo (Fig. 2; Supplementary Tables S7 and S8).

**Figure 2.**
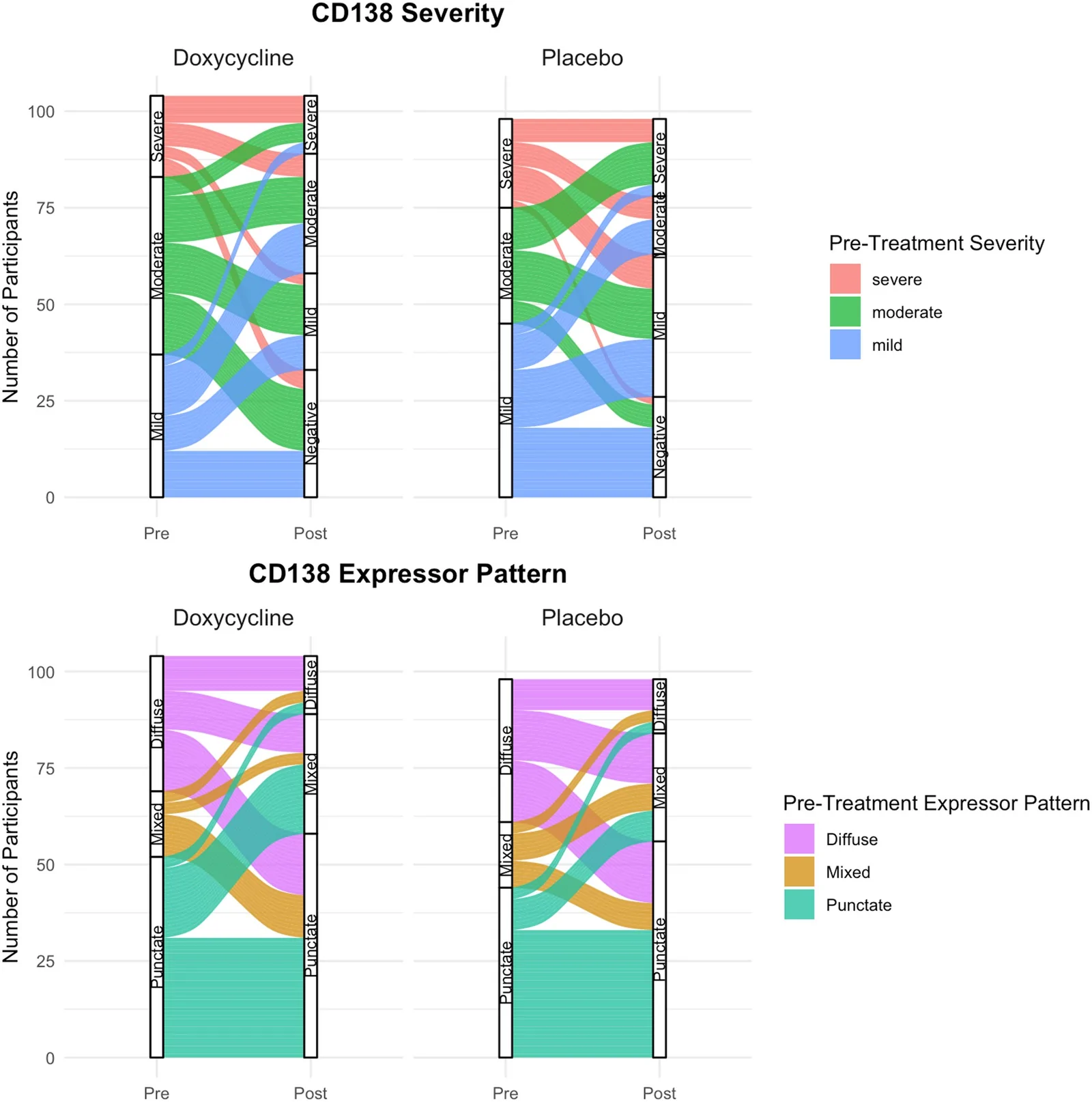
**A Sankey diagram indicating change in CD138 severity and expressor pattern pre and post doxycycline.** There was no difference in the transition status between each CE severity category (severe, moderate, mild, negative) pre and post treatment in the doxycycline group (*P* = 0.264, Fisher’s exact). The expressor pattern transition statuses from pre-treatment to post-treatment showed no difference (*P* = 0.572, Chi square).

## Discussion

This is the first randomised placebo-controlled trial of Doxycycline in women with a history of RM and CE as defined by all CD138+ stromal cells, not just those that were morphologically plasma cells. We found no difference in our primary outcome of live birth or ongoing pregnancy (aRR 1.02; CrI 0.85–1.21). There were several strands of evidence supporting futility. The low probability of moderate effect on the primary outcome measure of cumulative live birth rate (*Prob.* = 0.34). In terms of secondary outcome measures, Doxycycline had no effect on time to conception or time to 24 weeks gestation. In the first pregnancies after treatment, there were no differences in the live birth or miscarriage rates in those randomised to Doxycycline or placebo. In an exploratory analysis women negative for CD138+ CE, there were significantly fewer live births and more miscarriages than in the placebo group (aRR 0.73; CrI 0.56–0.91, aRR 1.47; CrI 1.14–1.93). CD138+ CE severity varied after treatment with both Doxycycline and placebo indicating significant endometrial inter-cycle variation independent of Doxycycline. In a linked mechanistic study, we found that the CD138+ CE test was positive because of delayed endometrial maturation rather than our hypothesised dysbiosis in the microbiome (Odendaal *et al.*, 2026).

Previous uncontrolled non-randomised studies reported improvements in histological CE (Johnston-MacAnanny *et al.*, 2010; McQueen *et al.*, 2014; Cicinelli *et al.*, 2015; Kitaya *et al.*, 2017; Huang *et al.*, 2020; Song *et al.*, 2021), time to clinical pregnancy (Mitter *et al.*, 2021) and live birth (McQueen *et al.*, 2014; Huang *et al.*, 2020; Liu *et al.*, 2022) with antibiotics. The absence of adequately controlled studies has prevented the observation of the inter-cycle variation in CE seen in our placebo group, as well as the comparison of clinical outcomes with those receiving placebo. The better live birth rate in CD138+ CE positive women on placebo compared to CD138+ CE negative could be accounted for by the inclusion criteria including all CD138+ CE positive stromal cells detecting delayed endometrial maturation rather than infection (Odendaal *et al.*, 2026). This delayed maturation could have detected a defective luteal phase responsive to progesterone (Practice Committee of the American Society for Reproductive Medicine, 2012b). Progesterone was administered within the trial in pregnancy for threatened miscarriage possibly correcting this defect (Coomarasamy *et al.*, 2019) as recorded in the concomitant medications table (Supplementary Table S5). We did not collect concomitant medication from the CD138 CE negative group but its use was prevalent amongst recruiting clinicians. Given this was not primarily assessed within the trial however, this remains a mechanistic hypothesis requiring further investigation.

A primary strength of this study was the selection of cumulative live birth rate as the primary outcome measure. This patient-centred approach ensured that a live birth following an initial miscarriage of an abnormal pregnancy was still recorded as a successful outcome. These results were further validated by analysing the conventional live birth rate in the first pregnancy post-intervention. Methodologically, the trial utilised a robust triple-blind, placebo-controlled framework. Rigour was maintained through a published protocol, oversight committees and an efficient adaptive Bayesian design featuring pre-specified stopping measures. The Bayesian analysis offered the added benefit of getting confident probabilistic statements for the entire spectrum of evidence about the primary outcome, in a highly interpretable format, even in this case of an early terminating clinical trial (Spiegelhalter *et al.*, 2004; Yin, 2012; Odendaal *et al.*, 2021; Wendelberger and Lewis, 2023).

Despite the rigorous framework the trial has some limitations. There were challenges in recruitment that contributed to the funder decision to stop the trial. However, the original adaptive Bayesian design allowed for stopping due to futility at interim analyses. Recruitment was complicated by the fact that the intervention was marketed and accessible. However, a similar number of women in each group received concomitant medication to prevent miscarriage. A higher-than-expected prevalence rate of 71% was found for CE. This prevalence was within the previously reported ranges (Cicinelli *et al.*, 2014; McQueen *et al.*, 2014; Liu *et al.*, 2018; Moreno *et al.*, 2018) and was a consequence of a permissive cellular level inclusion criteria (low threshold for CE diagnosis). A systematic review also demonstrated a broad diagnostic criterion to be associated with a higher incidence of CE illustrating the variability in which CD138 cells are included in CE diagnosis (Huang *et al.*, 2020). This trial was done with strict adherence to a protocol and statistical analysis plan. This prespecified plan was to categorise the CD138 staining into mild (5–20 CD138^+^/10 mm^2^), moderate (21–200 CD138^+^/10 mm^2^) and severe (>200 CD138^+^/10mm^2^), then to do a sensitivity analysis. The sensitivity analysis was conducted to assess whether the results were altered by the diagnostic inclusion criteria, and we did not find an interaction between severity group and treatment group. The exact number in each group is given in Supplementary Table S2.

In a post hoc analysis, we have also presented data for the trial separated by punctate staining similar to the plasma cell morphology described by Liu *et al.* (2018). There is no signal of a positive effect of doxycycline in this subgroup (Supplementary Table S9).

Since the overall effect estimate is close to zero for there to be a clear benefit in a sub-group, sizeable enough to be clinically important, it is highly likely that doxycycline would have had a detrimental effect in the other sub-group. This has a low level of clinical plausibility. Our overall conclusions only apply to a population defined by presence of CD138. Nevertheless, based on our data, it is highly unlikely that a true positive effect is present in any sub-group. In the absence of fresh RCT showing a benefit in such a sub-group, the continued use of doxycycline to prevent recurrent first trimester miscarriage may be difficult to justify.

Trial medication was started on day one of the cycle given for 14 days whilst the endometrium is regenerating. The second biopsies were taken 10 (±4) days after the medication stopped on day 24(±4) of the cycle; hence, our second biopsies should have detected a treatment effect. However, as the second biopsies occurred within the treatment menstrual cycle, it is also possible that a difference in CE would have been detected had the second biopsy occurred in a subsequent cycle after menstruation and regeneration of the endometrium. Our data challenge the clinical practise of repeating the biopsy then trying a different antibiotic, as the apparent ‘cure’ rate was similar in the Doxycycline (32%) and the placebo groups (27%) *P* = 0.44 (Supplementary Table S5). A secondary outcome of doxycycline giving a significant reduction in ectopic pregnancy (aRR 76.7; CrI 0.38–241) needs interpreting with extreme caution due to the wide credible intervals. It is, nevertheless, possible that the doxycycline did treat low-grade tubal infection preventing ectopic pregnancy, or it may be a chance observation because of multiple comparisons. This trial was not designed to show any effect on second trimester miscarriages. We do not know if any participating women might, in addition to being CD138+ also had polyps or sub-mucous fibroids that might have reduced chances of successful pregnancy in both randomised groups.

## Conclusions

In this first placebo-controlled trial, the totality of our findings does not support the use of doxycycline in the management of CE as defined by CD138 staining in stromal cells in recurrent miscarriage. Doxycycline compared to placebo did not change the live birth, miscarriage rates, time to conception or the endometrium. We used a broad inclusion criteria of all stromal cells stained with CD138 and a sensitivity analysis increasing the diagnostic cut-off did not alter these findings.

## Supplementary Material

deag133_Supplementary_materials_and_methods

deag133_Supplementary_Figure_S1

deag133_Supplementary_Figure_S2

deag133_Supplementary_Figure_S3

deag133_Supplementary_Figure_S4

deag133_Supplementary_Figure_S5

deag133_Supplementary_Table_S1

deag133_Supplementary_Table_S2

deag133_Supplementary_Table_S3

deag133_Supplementary_Table_S4

deag133_Supplementary_Table_S5

deag133_Supplementary_Table_S6

deag133_Supplementary_Table_S7

deag133_Supplementary_Table_S8

deag133_Supplementary_Table_S9

## Data Availability

Anonymised participant data are available on reasonable request to the data access team in the Warwick CTU wctudataaccess@warwick.ac.uk. For full trial protocol and statistical analysis plan, visit https://warwick.ac.uk/fac/sci/med/research/ctu/trials/cerm/
