# Supplementary material for: A double-blind multi-centre randomised placebo-controlled trial of doxycycline in women with recurrent miscarriage and chronic endometritis defined by CD138 positive stromal cells
Source: Hum Reprod. 2026 Aug 20;41(10):1661–71. doi: 10.1093/humrep/deag133 (PMC13635465; doi:10.1093/humrep/deag133)
Supplement: Supplementary file 1 [file deag133_supplementary_figure_s1.pdf]

Human Reproduction, 2026, 1–1
https://doi.org/10.1093/humrep/deag133
Supplementary Data

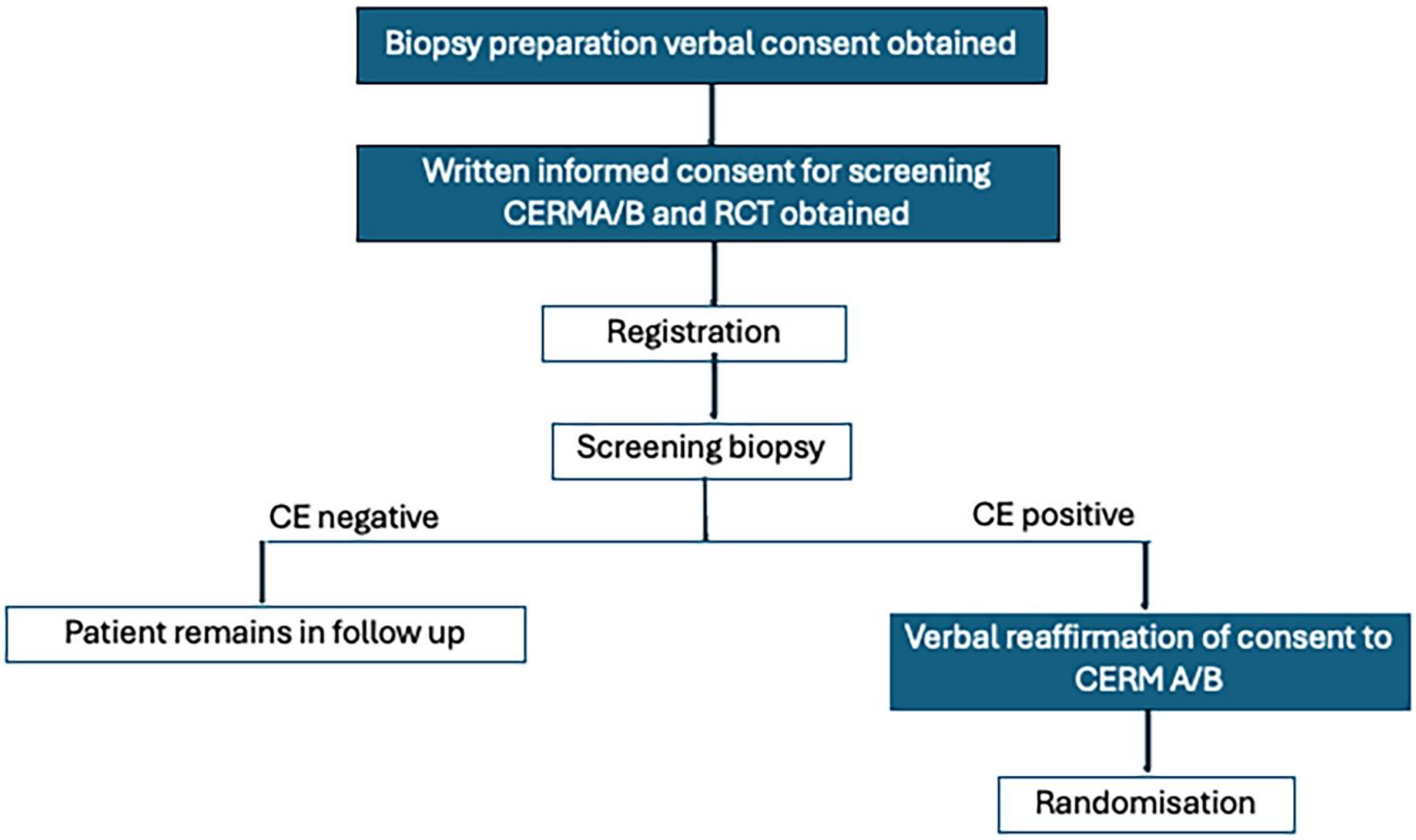

Supplementary Figure S1. **Consent overview.** A three-stage consent process was used, women gave verbal consent to receive, information about the trial, ovulation kits and condoms. They gave written consent to participate in the trial at the screening biopsy, and written consent was confirmed at the time of randomisation. CERM A was the clinical study. CERM B was the mechanistic study.
