# Supplementary material for: A double-blind multi-centre randomised placebo-controlled trial of doxycycline in women with recurrent miscarriage and chronic endometritis defined by CD138 positive stromal cells
Source: Hum Reprod. 2026 Aug 20;41(10):1661–71. doi: 10.1093/humrep/deag133 (PMC13635465; doi:10.1093/humrep/deag133)
Supplement: Supplementary file 3 [file deag133_supplementary_figure_s3.pdf]

Human Reproduction, 2026, 1–1
https://doi.org/10.1093/humrep/deag133
Supplementary Data

human reproduction
OXFORD

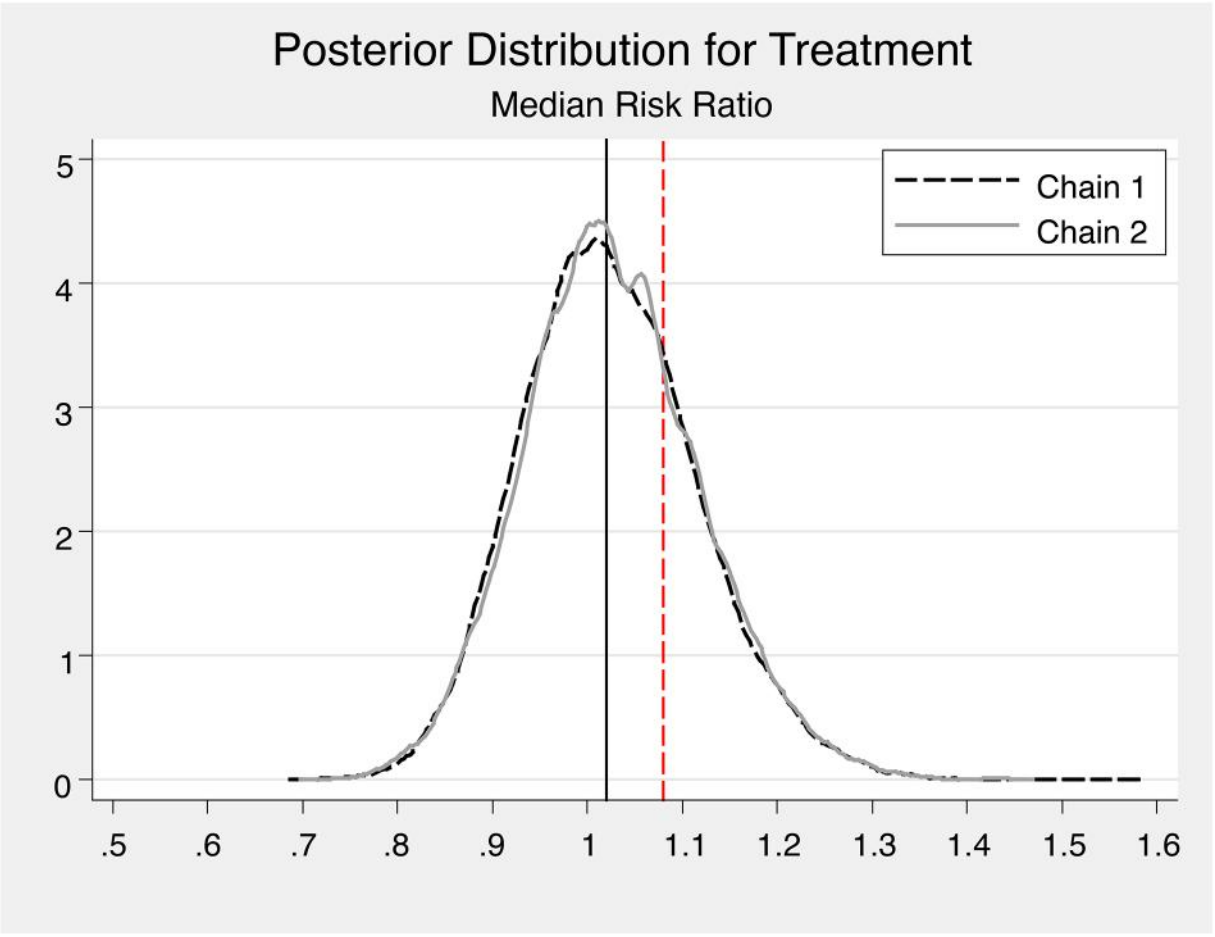

Supplementary Figure S3. Posterior distribution for treatment effect on primary outcome. The graph displays the posterior distribution of Risk Ratio for the Doxycycline. In the Y-axis, the Density is displayed whereas the X-axis provides a ratios between 0.5 and 1.6, with ratio 1 indicating no difference between the two arms, 1.036 the model- adjusted median ratio (black (solid) vertical line) and 1.16 the anticipated theoretical risk ratio (red (dashed) vertical line) that would indicate 8% or higher increase in deliveries favouring Doxycycline. The two MCMC chains used indicate good model convergence.
