# Supplementary material for: A double-blind multi-centre randomised placebo-controlled trial of doxycycline in women with recurrent miscarriage and chronic endometritis defined by CD138 positive stromal cells
Source: Hum Reprod. 2026 Aug 20;41(10):1661–71. doi: 10.1093/humrep/deag133 (PMC13635465; doi:10.1093/humrep/deag133)
Supplement: Supplementary file 5 [file deag133_supplementary_figure_s5.pdf]

Human Reproduction, 2026, 1–1
https://doi.org/10.1093/humrep/deag133
Supplementary Data

human reproduction
OXFORD

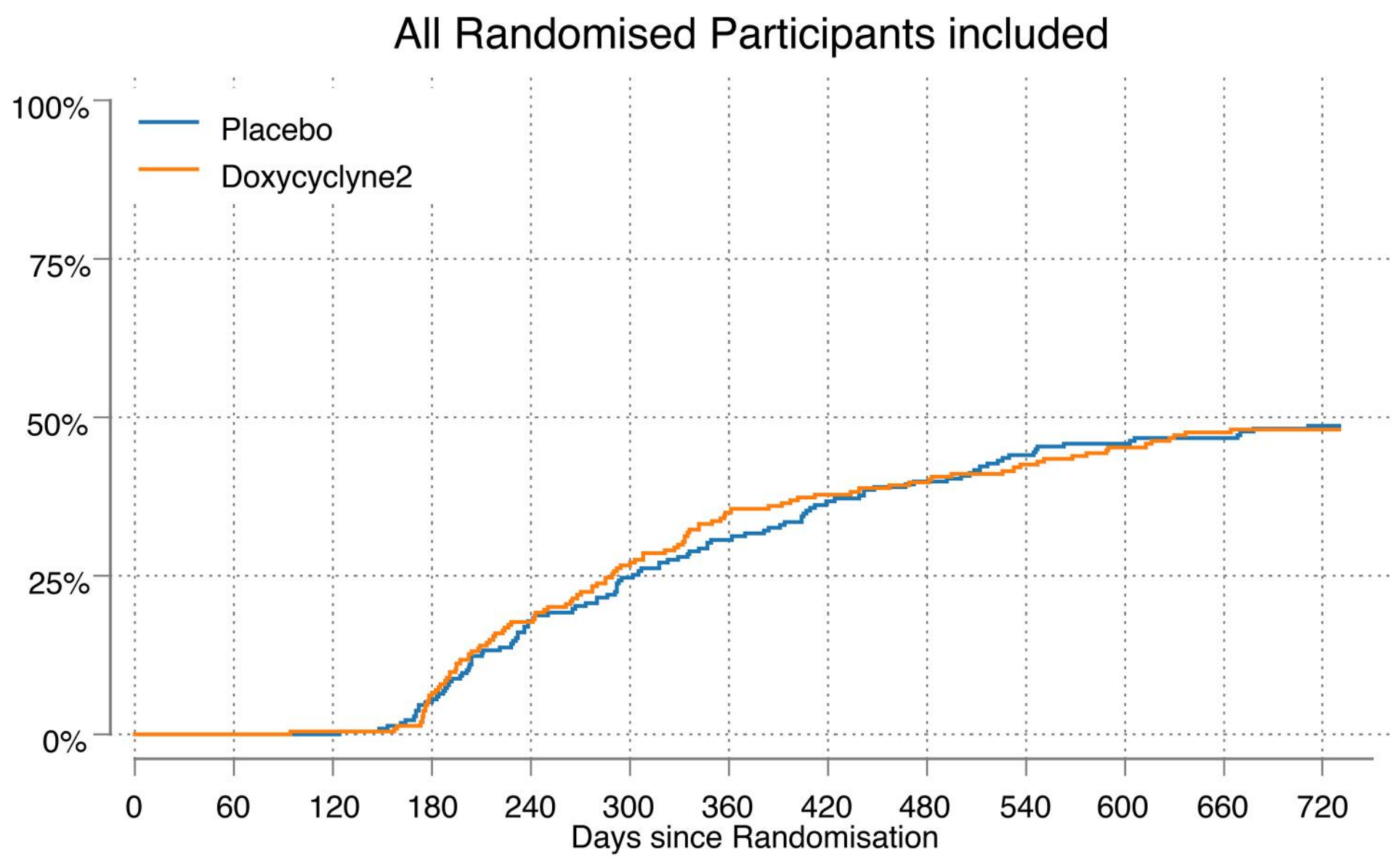

Number at risk

arm1	218	218	218	207	179	164	151	138	131	122	118	116	112
arm2	214	214	213	201	176	157	139	133	129	123	117	112	111

Supplementary Figure S5. **Cumulative time to 24 weeks gestation.**
