# Supplementary material for: A double-blind multi-centre randomised placebo-controlled trial of doxycycline in women with recurrent miscarriage and chronic endometritis defined by CD138 positive stromal cells
Source: Hum Reprod. 2026 Aug 20;41(10):1661–71. doi: 10.1093/humrep/deag133 (PMC13635465; doi:10.1093/humrep/deag133)
Supplement: Supplementary file 6 [file deag133_supplementary_materials_and_methods.pdf]

Human Reproduction, 2026, 1–1
https://doi.org/10.1093/humrep/deag133
Supplementary Data

human reproduction

OXFORD

Supplementary materials and methods

CD138 immunohistochemistry methods

This was caried out in a single laboratory experienced in endometrial biopsy analysis that followed United Kingdom Accreditation Service standards. The lead site undertook biopsies from patients whose local clinic were not able to take timed endometrial biopsies. Hence, patients either travelled to the lead site and had their biopsies taken and put in formalin then or had biopsies taken at a remote site and posted to the lead site in formalin. Freshly collected endometrial biopsy samples were placed in 10% neutral buffered formalin for at least 24 h with a maximum time of 7 days. Timing between taking the biopsy and processing was closely monitored to ensure all samples were treated similarly. Formalin-fixed samples were embedded using Shandon Excelsior ES Tissue processor 96 (ThermoFisher; Waltham, Massachusetts, United States) in Surgipath® Formula 'R'™ paraffin. Then, 3 µm sections were taken and baked overnight at 60°C on glass slides. The Roche Ventana Discovery XT Autostainer (Roche, F. Hoffmann-La Roche Ltd. Basel, Switzerland) was used, for immunohistochemistry. Manufacturer's instructions were followed as per Roche certificate of analysis. In outline, for antigen retrieval, CC1, which is a high pH buffer was used for 40 min at 100°C. Slides were then incubated with predilute B-A38 CD138 antibody at 37°C for 44 min. Visualisation was with OptiView DAB IHC Detection Kit/760-700 incubated for 8 min with linker and 8 min with polymer at 36°C. Chromogen was with OptiView DAB IHC Detection Kit/760-700 for 8 min at 36°C enhanced with CuSO4. Each staining run had a positive control of tonsillar tissue and negative control of tonsillar tissue stained by a monoclonal antibody not directed against a known epitope in human tissue. Slides were coverslipped. Images of the slides underwent digitisation on a 3D Histech P150 slide scanner using a 20× objective lens.

Slides were reviewed using Case Viewer V2.2 (3DHISTECH Ltd, Budapest, Hungary). Entire sections underwent review at 20× magnification and positive cells counted. Tissue surface was in addition calculated to determine the $CD138^+$ cell density per 10 mm^2. Samples were stratified into four categories to further aid prognostic analysis: negative (<5 $CD138^+/10\ mm^2$), mild (5–20 $CD138^+/10\ mm^2$), moderate (21–200 $CD138^+/10\ mm^2$) and severe (>200 $CD138^+/10\ mm^2$). This classification is similar to that reported by others (De Smet *et al.*, 2024). Staining patterns were characterised as punctate were >50% of staining was punctate and similarly for diffuse. Where equal proportions of pattern were present, a mixed pattern was noted.

A small group of five observers all trained on a test set of slides, their inter-observer correlation was good (R 0.64-0.84, $P < 0.01$) inter rater agreement was moderate to strong Cohen Kappa (0.42–0.64). This was consistent with others.

Quality assurance processes included, borderline samples were assessed by two independent assessors and endometrial samples of insufficient size were excluded and women were offered a second biopsy in another cycle and random sample assessment was validated by external expert.

There were three layers of external quality control, an expert in reproductive immunology from the trial management committee who assessed a random sample of images of samples stained with immunohistochemistry for CD138 and the detailed staining protocol was ratified by the chair of the trial steering committee with expertise in translational reproductive medicine and a third international expert in CE and RPL from the data monitoring committee.

Reference

De Smet S, Mackens S, Blockeel C, Tournaye H, Forsyth R, Waelput W. P-322 Development of a CD138-based histopathological scoring system to diagnose chronic endometritis. *Human Reproduction* 2024; **39**:deae108.687. https://doi.org/10.1093/humrep/deae108.687
