# Supplementary material for: A double-blind multi-centre randomised placebo-controlled trial of doxycycline in women with recurrent miscarriage and chronic endometritis defined by CD138 positive stromal cells
Source: Hum Reprod. 2026 Aug 20;41(10):1661–71. doi: 10.1093/humrep/deag133 (PMC13635465; doi:10.1093/humrep/deag133)
Supplement: Supplementary file 7 [file deag133_supplementary_table_s1.pdf]

Human Reproduction, 2026, 1–1
https://doi.org/10.1093/humrep/deag133
Supplementary Data

human reproduction

OXFORD

Supplementary Table S1. Recruiting sites.

Site name	Frequency	Percent
Addenbrooke's Hospital	4	0.54
Birmingham Women's Hospital	2	0.27
Burnley General Hospital	32	4.34
Calderdale Royal Hospital	5	0.68
Darlington Memorial Hospital	1	0.14
Dumfries & Galloway Royal Infirmary	4	0.54
Heartlands Hospital	4	0.54
Ipswich Hospital	13	1.76
King's College Hospital	8	1.09
Liverpool Women's Hospital NHS Foundation Trust	13	1.76
Norfolk and Norwich University Hospital	13	1.76
Oxford University Hospital	16	2.17
Queen Alexandra Hospital	4	0.54
Royal United Hospital Bath	13	1.76
Singleton Hospital	6	0.81
St Richard's Hospital	19	2.58
Sunderland Royal Hospital	2	0.27
The Princess Royal Hospital	1	0.14
The Royal London Hospital	5	0.68
The James Cook University Hospital	9	1.22
University College London Hospitals NHS Trust	3	0.41
University Hospital of North Durham	4	0.54
University Hospitals Coventry and Warwickshire NHS Trust*	538	73.00
West Middlesex Hospital	6	0.81
Worthing Hospital	6	0.81
Wrightington Wigan and Leigh NHS Foundation Trust	6	0.81
Total	737	100.00

* Lead site.
