# Supplementary material for: A double-blind multi-centre randomised placebo-controlled trial of doxycycline in women with recurrent miscarriage and chronic endometritis defined by CD138 positive stromal cells
Source: Hum Reprod. 2026 Aug 20;41(10):1661–71. doi: 10.1093/humrep/deag133 (PMC13635465; doi:10.1093/humrep/deag133)
Supplement: Supplementary file 8 [file deag133_supplementary_table_s2.pdf]

Human Reproduction, 2026, 1–1
https://doi.org/10.1093/humrep/deag133
Supplementary Data

human reproduction

OXFORD

Supplementary Table S2. Recruitment of patients at the lead site (University Hospitals Coventry and Warwickshire).

Region	Number of patients N = 538	Percentage
Local, Coventry and Warwickshire	124	23%
Other West Midlands areas	168	32%
East Midlands	71	13%
South West	61	11%
North West	36	7%
South East	18	3%
North East	17	3%
East Anglia	17	3%
London	13	2%
Wales	7	1%
Scotland	3	0.5%
Northern Ireland	3	0.5%

The main site recruited 73% of patients but received referrals from across the UK for screening and randomisation. At the lead site 77% attended for trial procedures only and other care continued at the referring hospital.
