# Supplementary material for: A double-blind multi-centre randomised placebo-controlled trial of doxycycline in women with recurrent miscarriage and chronic endometritis defined by CD138 positive stromal cells
Source: Hum Reprod. 2026 Aug 20;41(10):1661–71. doi: 10.1093/humrep/deag133 (PMC13635465; doi:10.1093/humrep/deag133)
Supplement: Supplementary file 9 [file deag133_supplementary_table_s3.pdf]

Human Reproduction, 2026, 1–1
https://doi.org/10.1093/humrep/deag133
Supplementary Data

human reproduction
OXFORD

Supplementary Table S3. Baseline biopsy results.

	Placebo	Doxycycline	Biopsy negative
Number (%)	219	219	208
CE status			
Mild	79 (36.1)	79 (36.1)	0
Moderate	73 (33.1)	78 (35.6)	0
Severe	67 (30.6)	62 (28.3)	0
CE pattern			
Punctate	76 (34.7)	102 (46.6)	196 (94.2)
Diffuse	101 (46.1)	87 (39.7)	6 (2.9)
Mixed	42 (19.2)	30 (14.0)	6 (2.9)
P type cells			
Yes	123 (56.2)	121 (55.3)	136 (65.3)
No	96 (43.8)	98 (44.7)	72 (34.6)

CE, chronic endometritis.
