# Supplementary material for: A double-blind multi-centre randomised placebo-controlled trial of doxycycline in women with recurrent miscarriage and chronic endometritis defined by CD138 positive stromal cells
Source: Hum Reprod. 2026 Aug 20;41(10):1661–71. doi: 10.1093/humrep/deag133 (PMC13635465; doi:10.1093/humrep/deag133)
Supplement: Supplementary file 10 [file deag133_supplementary_table_s4.pdf]

Human Reproduction, 2026, 1–1
https://doi.org/10.1093/humrep/deag133
Supplementary Data

human reproduction
OXFORD

Supplementary Table S4. Treatment compliance post-randomisation.

Compliance	Placebo n = 219	Doxycycline n = 219
**Took all medication**	194	199
Missed 1–3 tablets but completed course	11	5
Missed 1–3 tablets	3	5
Met compliance criteria for taking IMP	208 (95%)	209 (95%)
**Did not take any IMP**		
Did not collect	8	8
Unknown compliance	3	2
**Additional externally sourced doxycycline**		
Before pregnancy for CE	4	4
Before first pregnancy for other indications	1	2
After first pregnancy for acute endometritis	4	2

CE, chronic endometritis; IMP, Investigational Medicinal Product.
