# Supplementary material for: A double-blind multi-centre randomised placebo-controlled trial of doxycycline in women with recurrent miscarriage and chronic endometritis defined by CD138 positive stromal cells
Source: Hum Reprod. 2026 Aug 20;41(10):1661–71. doi: 10.1093/humrep/deag133 (PMC13635465; doi:10.1093/humrep/deag133)
Supplement: Supplementary file 11 [file deag133_supplementary_table_s5.pdf]

Human Reproduction, 2026, 1–1
https://doi.org/10.1093/humrep/deag133
Supplementary Data

human reproduction OXFORD

Supplementary Table S5. Concomitant medications in pregnancy.

	Placebo N = 219	Doxycycline N = 219
Progesterone	42 (19%)	35 (16%)
Aspirin	24 (11%)	24 (11%)
Low molecular weight heparin	4 (2%)	4 (2%)
Prednisolone	1(0.5%)	1(0.5%)
Hydroxychloroquine	3 (1%)	4 (2%)
