# Supplementary material for: A double-blind multi-centre randomised placebo-controlled trial of doxycycline in women with recurrent miscarriage and chronic endometritis defined by CD138 positive stromal cells
Source: Hum Reprod. 2026 Aug 20;41(10):1661–71. doi: 10.1093/humrep/deag133 (PMC13635465; doi:10.1093/humrep/deag133)
Supplement: Supplementary file 12 [file deag133_supplementary_table_s6.pdf]

Human Reproduction, 2026, 1–1
https://doi.org/10.1093/humrep/deag133
Supplementary Data

human reproduction OXFORD

Supplementary Table S6. Type of miscarriage experienced.

Miscarriage type	Randomised to Placebo	Randomised to Doxycycline	CE negative
First pregnancy			
Biochemical loss	26	17	38
No foetus in gestion sac	5	4	4
Previously viable pregnancy	21	22	14
Other	3	11	9
First pregnancy total	55	54	65
Second pregnancy			
Biochemical loss	7	7	
No foetus in gestion sac	1	0	
Previously viable pregnancy	3	2	
Other	1	2	
Second pregnancy total	12	11	
Third pregnancy			
Biochemical loss	1	2	
No foetus in gestion sac	0	0	
Previously viable pregnancy	1	0	
Other	0	0	
Third pregnancy total	2	2	
Fourth pregnancy			
Biochemical loss	0	1	
No foetus in gestion sac	0	0	
Previously viable pregnancy	0	0	
Other	0	0	
Fourth pregnancy total	0	1	

CE, chronic endometritis.
Further exploration of the women who suffered an ectopic pregnancy found that one woman in each arm had an ectopic pregnancy prior to randomisation, six ectopic pregnancies occurred in women whose pregnancy loss history was of miscarriages only.
