# Supplementary material for: A double-blind multi-centre randomised placebo-controlled trial of doxycycline in women with recurrent miscarriage and chronic endometritis defined by CD138 positive stromal cells
Source: Hum Reprod. 2026 Aug 20;41(10):1661–71. doi: 10.1093/humrep/deag133 (PMC13635465; doi:10.1093/humrep/deag133)
Supplement: Supplementary file 13 [file deag133_supplementary_table_s7.pdf]

Human Reproduction, 2026, 1–1
https://doi.org/10.1093/humrep/deag133
Supplementary Data

human reproduction OXFORD

Supplementary Table S7. CE transition status.

Doxycycline

		Biopsy B				
		**Negative**	**Mild**	**Moderate**	**Severe**	**Total**
Biopsy A	Mild	12	9	13	3	37
	Row %	32.4	24.3	35.1	8.1	
	Moderate	16	13	12	5	46
	Row %	34.8	28.3	26.1	10.9	
	Severe	5	3	6	7	21
	Row %	23.8	14.3	28.6	33.3	
	Total	33	25	31	15	104

Placebo

		Biopsy B				
		**Negative**	**Mild**	**Moderate**	**Severe**	**Total**
Biopsy A	Mild	18	15	9	3	45
	Row %	40.0	33.3	20.0	6.7	
	Moderate	6	13	0	11	30
	Row %	20.0	43.3	0.0	36.7	
	Severe	2	9	6	6	23
	Row %	8.7	39.1	26.1	26.1	
	Total	26	37	15	20	98

CE, chronic endometritis.

One hundred and four participants randomised to the doxycycline arm and 98 participants randomised to the placebo arm had a repeat biopsy following treatment; 31.7% (33/104) of the doxycycline group and 26.5% (26/98) of the placebo group tested negative for CE, and this difference was not statistically significant ($P = 0.44$, Fisher's exact). Of the 104 allocated to the doxycycline arm, 35.6% (37/104) were classified as mild CE, 44.2% (46/104) had moderate CE and 20.2% (21/104) had severe CE. Of the 98 participants allocated to the placebo arm, 45.9% (45/98) had mild CE, 30.6% (30/98) had moderate CE and 23.5% (23/98) had severe CE. There was no difference in the transition status between each CE severity category (severe, moderate, mild and negative) pre- and post-treatment in the doxycycline group ($P = 0.264$, Fisher's exact). There was a statistical difference between CE severity categories pre- and post-treatment in the placebo group ($P = 0.001$, Fisher's exact); however, this was not considered clinically meaningful, as the change reflected reclassification of initially moderate cases changing to severe, mild or negative categories.
