# Supplementary material for: A double-blind multi-centre randomised placebo-controlled trial of doxycycline in women with recurrent miscarriage and chronic endometritis defined by CD138 positive stromal cells
Source: Hum Reprod. 2026 Aug 20;41(10):1661–71. doi: 10.1093/humrep/deag133 (PMC13635465; doi:10.1093/humrep/deag133)
Supplement: Supplementary file 14 [file deag133_supplementary_table_s8.pdf]

Human Reproduction, 2026, 1–1
https://doi.org/10.1093/humrep/deag133
Supplementary Data

human reproduction

OXFORD

Supplementary Table S8. CE expressor pattern transition status.

Doxycycline

		Biopsy B			
		**Punctate**	**Mixed**	**Diffuse**	**Total**
Biopsy A	Punctate	31	18	3	52
	%	59.6	34.6	5.8	
	Mixed	11	3	3	17
	%	64.7	17.6	17.6	
	Diffuse	16	10	9	35
	%	45.7	28.6	25.7	
	Total	58	31	15	104

Placebo

		Biopsy B			
		**Punctate**	**Mixed**	**Diffuse**	**Total**
Biopsy A	Punctate	33	8	3	44
	%	75.0	18.2	6.8	
	Mixed	7	7	3	17
	%	41.2	41.2	17.6	
	Diffuse	16	13	8	37
	%	43.2	35.1	21.6	
	Total	56	28	14	98

CE, chronic endometritis.
The samples were analysed by expression pattern of CD138 immunostaining. Before treatment, the doxycycline group demonstrated 50% (52/104) were of a punctate expression, 16.3% (17/104) were mixed and 33.7% (35/104) showed a diffuse staining pattern. In the placebo group, 44.9% (44/98) were punctate, 17.3% (17/98) were mixed and 37.8% (37/98) were diffuse. The expressor pattern transition statuses from pre-treatment to post-treatment showed no difference ($P = 0.572$, Chi square).
