# Supplementary material for: A double-blind multi-centre randomised placebo-controlled trial of doxycycline in women with recurrent miscarriage and chronic endometritis defined by CD138 positive stromal cells
Source: Hum Reprod. 2026 Aug 20;41(10):1661–71. doi: 10.1093/humrep/deag133 (PMC13635465; doi:10.1093/humrep/deag133)
Supplement: Supplementary file 15 [file deag133_supplementary_table_s9.pdf]

Human Reproduction, 2026, 1–1
https://doi.org/10.1093/humrep/deag133
Supplementary Data

human reproduction

OXFORD

Supplementary Table S9. Pregnancy outcome according to pattern of staining.

		Total	Conceived	Cumulative primary out-come Livebirth + ongoing pregnancies
Punctate	placebo	76	62	42 (55%)
	doxycycline	101	63	45 (45%)
Diffuse	placebo	102	62	44 (43%)
	doxycycline	87	60	46 (53%)

© The Author(s) 2026. Published by Oxford University Press on behalf of European Society of Human Reproduction and Embryology.
This is an Open Access article distributed under the terms of the Creative Commons Attribution License (https://creativecommons.org/licenses/by/4.0/), which permits unrestricted reuse, distribution, and reproduction in any medium, provided the original work is properly cited.
Human Reproduction, 2026, 00, 1–26
https://doi.org/10.1093/humrep/deag133
Original Article
